# Rapid SNP Discovery and Genetic Mapping Using Sequenced RAD Markers

**DOI:** 10.1371/journal.pone.0003376

**Published:** 2008-10-13

**Authors:** Nathan A. Baird, Paul D. Etter, Tressa S. Atwood, Mark C. Currey, Anthony L. Shiver, Zachary A. Lewis, Eric U. Selker, William A. Cresko, Eric A. Johnson

**Affiliations:** 1 Institute of Molecular Biology, University of Oregon, Eugene, Oregon, United States of America; 2 Floragenex, Eugene, Oregon, United States of America; 3 The Center for Ecology and Evolutionary Biology, University of Oregon, Eugene, Oregon, United States of America; Washington University, United States of America

## Abstract

Single nucleotide polymorphism (SNP) discovery and genotyping are essential to genetic mapping. There remains a need for a simple, inexpensive platform that allows high-density SNP discovery and genotyping in large populations. Here we describe the sequencing of restriction-site associated DNA (RAD) tags, which identified more than 13,000 SNPs, and mapped three traits in two model organisms, using less than half the capacity of one Illumina sequencing run. We demonstrated that different marker densities can be attained by choice of restriction enzyme. Furthermore, we developed a barcoding system for sample multiplexing and fine mapped the genetic basis of lateral plate armor loss in threespine stickleback by identifying recombinant breakpoints in *F_2_* individuals. Barcoding also facilitated mapping of a second trait, a reduction of pelvic structure, by *in silico* re-sorting of individuals. To further demonstrate the ease of the RAD sequencing approach we identified polymorphic markers and mapped an induced mutation in *Neurospora crassa*. Sequencing of RAD markers is an integrated platform for SNP discovery and genotyping. This approach should be widely applicable to genetic mapping in a variety of organisms.

## Introduction

Genetic mapping of natural or induced genomic variation remains a powerful approach to understand the function of genes in a variety of biological processes. SNPs are the most abundant type of genetic marker and their high density makes them ideal for studying the inheritance of genomic regions [1]–[3]. However, current genotyping platforms often require a large investment to initially discover SNPs [4], [5] and to subsequently genotype them in a large number of individuals [5]–[7].

Genetic marker systems often identify SNPs through the disruption of restriction endonuclease recognition sites [8]–[10]. We previously developed a novel marker called restriction-site associated DNA (RAD), which are short fragments of DNA adjacent to each instance of a particular restriction enzyme recognition site. Hybridization of RAD tags to DNA microarrays allows the parallel screening of thousands of polymorphic markers to map natural variation and induced mutations in diverse organisms [11]–[13]. Although powerful and widely applicable, microarray-based RAD techniques can only assay a fraction of segregating polymorphisms.

Here we describe a significant advance in the RAD genotyping platform through the use of next-generation sequencers that allows comprehensive polymorphism data to be utilized. The massively parallel and multiplexed sample sequencing of RAD tag libraries facilitates the rapid discovery of thousands of SNPs and high-throughput genotyping of large populations. Sequenced RAD tags have several attractive features for genetic mapping. First, RAD tags create a reduced representation of the genome, allowing oversequencing of the nucleotides next to restriction sites and detection of SNPs. Second, a suitable number of markers for an application can be selected by choice of restriction enzyme, and the number of markers can be increased almost indefinitely by using additional enzymes. Third, the approach is amenable to genotyping pooled populations for bulk segregant analysis and also multiplexed genotyping of individuals for fine-scale mapping. Here we demonstrate the versatile and widely applicable nature of this high-throughput genetic mapping platform by performing bulk segregant and fine mapping of previously mapped traits in threespine stickleback, as well as bulk segregant mapping of a new induced mutation in *Neurospora crassa*.

## Results

### RAD marker generation for sequencing

**Figure 1:**
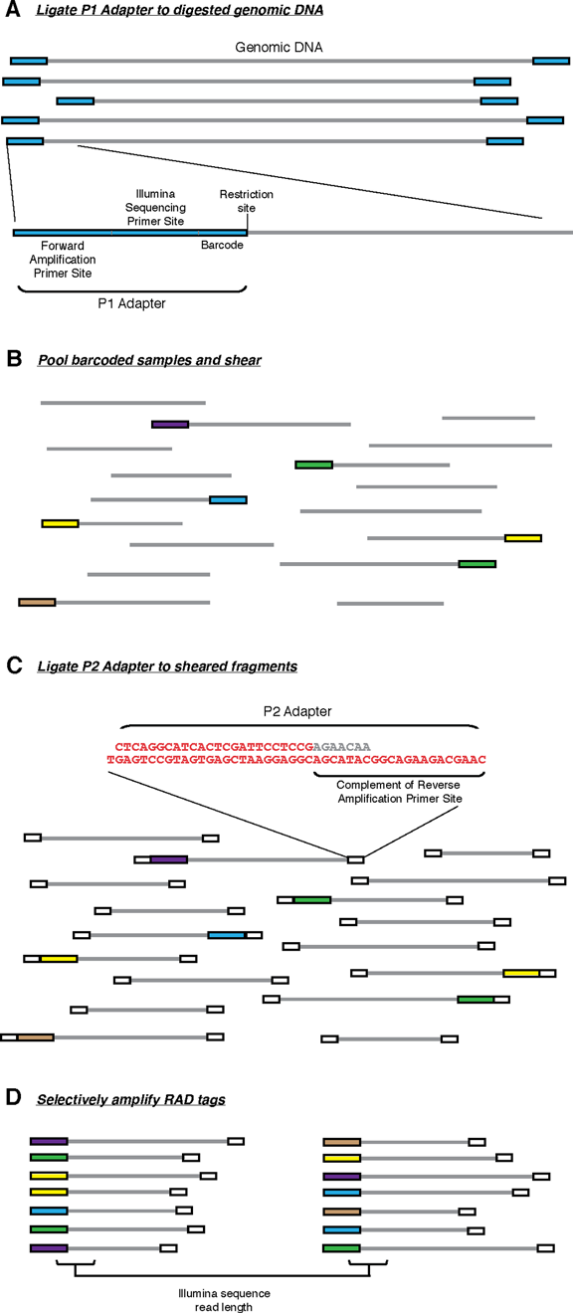
RAD marker generation. (A) Genomic DNA was digested with a restriction enzyme and the P1 adapter was ligated to the fragments. The P1 adapter contains a forward amplification primer site, an Illumina sequencing primer site, and a barcode (colored boxes represent P1 adapters with different barcodes). (B) Adapter-ligated fragments were combined (if multiplexing), sheared and (C) ligated to a second adapter (P2, white boxes). The P2 adapter is a divergent “Y” adapter, containing the reverse complement of the reverse amplification primer site preventing amplification of genomic fragments lacking a P1 adapter. (D) RAD tags, which have a P1 adapter, will be selectively and robustly enriched.

We developed new RAD tag generation and typing methods that greatly simplify and enhance genetic analysis through the use of an Illumina Genome Analyzer sequencer and the incorporation of nucleotide barcodes for sample tracking (Fig. 1). Genomic DNA was digested with a restriction enzyme and an adapter (P1) was ligated to the fragment's overhanging ends (Fig. 1A). This adapter contains forward amplification and Illumina sequencing primer sites, as well as a nucleotide barcode 4 or 5 bp long for sample identification. To reduce erroneous sample assignment due to sequencing error, all barcodes differ by at least two nucleotides. The adapter-ligated fragments were subsequently pooled, randomly sheared, and size-selected (Fig. 1B). DNA was then ligated to a second adapter (P2), a Y adapter [14] that has divergent ends (Fig. 1C). The reverse amplification primer is unable to bind to P2 unless the complementary sequence is filled in during the first round of forward elongation originating from the P1 amplification primer. The structure of this adapter ensures that only P1 adapter-ligated RAD tags will be amplified during the final PCR amplification step (Fig. 1D).

### Bulk segregant analysis with sequenced RAD markers

**Figure 2:**
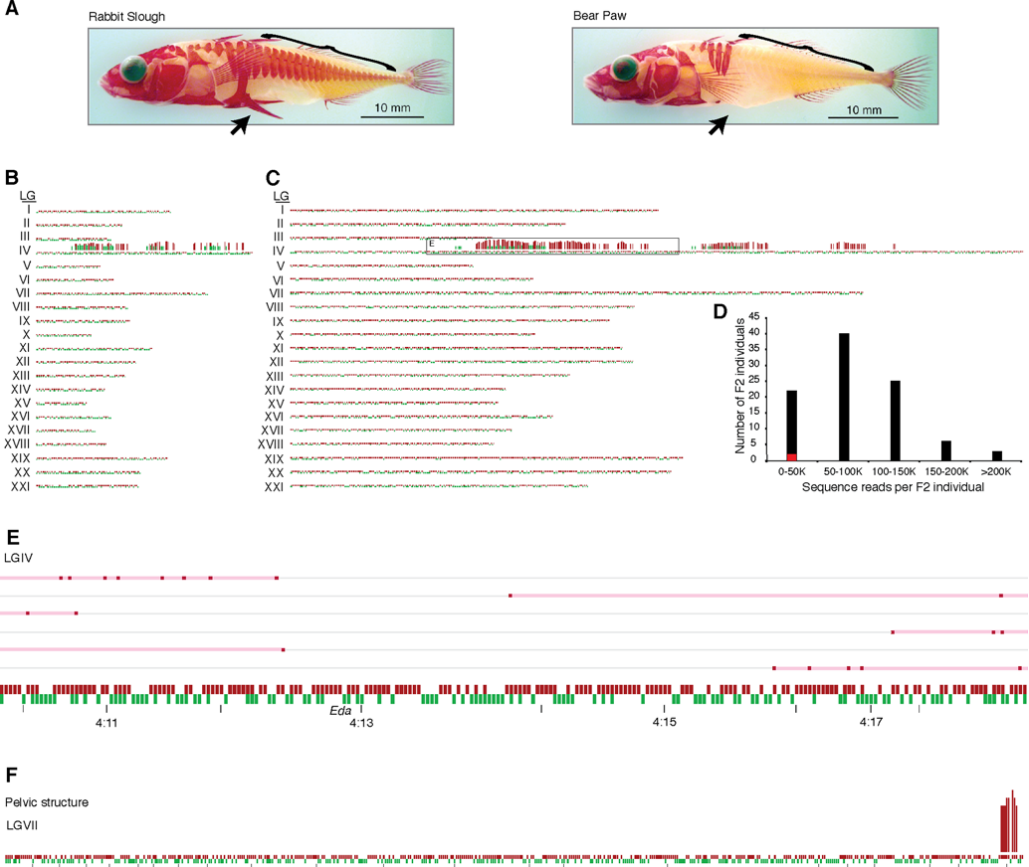
Sequenced RAD marker mapping. (A) A native saltwater stickleback population, Rabbit Slough (RS), have complete lateral plate armor (brackets) while these structures are absent in the derived, freshwater Bear Paw (BP) population. The freshwater fish also have a reduction in pelvic structure (arrow) compared to the oceanic population. These two phenotypes segregate independently in an *F_2_* mapping cross. Using *Sbf*I (B) or *EcoR*I (C), we mapped polymorphic RAD markers from RS (red) and BP (green) parental fish along the 21 stickleback linkage groups. The apparent size differences of the linkage groups between (B) and (C) reflect the fact that the *EcoR*I recognition sequence occurs more frequently than *Sbf*I. Red and green bars above the linkage groups are measures of lateral plate linkage in the *F_2_* progeny, indicating the number of tightly linked markers in the local region. (D) Sequence reads per barcoded *F_2_* individual used to create (C). Variable numbers of reads were obtained from each of the 96 individuals used in our analysis, reflecting different concentrations of starting DNA template. 68% of individuals had between 50 K and 150 K RAD tags sequenced (∼0.4–1.0× coverage of the ∼150 K tags present in the genome). Only 2 individuals had less than 10,000 reads (red). (E) A close-up of the boxed region from (C) showing recombination breakpoints in six informative low plate *F_2_* fish on LGIV. Black tick marks are 1 Mb apart in physical distance. (F) *F_2_* individuals were repooled *in silico* based on the pelvic structure phenotype (A, arrow). Linkage was determined as in (B, C), mapping the locus for a reduction in pelvic structure to the end of LGVII.

A major locus for the loss of bony lateral plates in multiple freshwater populations of threespine stickleback (Fig. 2A, brackets) has been previously mapped to linkage group (LG) IV [15], [16] and fine-mapped in some populations to the *Eda* locus [17]. We previously used RAD marker microarrays to remap this trait and found additional physically distinct regions of linkage along LGIV. To confirm the utility of Illumina sequencing for RAD tag analysis, as well as to determine if these additional regions of linkage are found in Alaskan populations other than the ones used in our original study [11], we analyzed an *F_2_* mapping cross between a different lake population with the low lateral plate phenotype (Bear Paw, BP) and the complete plate ancestral oceanic population (Rabbit Slough, RS).

As an initial test of RAD tag sequencing performance for genetic mapping, we created RAD tag libraries from parental RS and BP genomic DNA digested with *Sbf*I, which recognizes an 8-nucleotide sequence (CCTGCAGG). We sequenced 1.4 million RAD tags identified by the RS and BP parental barcodes and removed those that lacked an *Sbf*I site, yielding 608,000 BP and 760,000 RS RAD tags. The most error-prone portions of the sequences were removed and tags comprising the remaining 26 nucleotides were mapped unambiguously to 33,074 loci from the reference genome, with another 8,548 loci mapped with a single mismatch.

RAD markers polymorphic between the BP and RS parents were identified from this set of 41,622 RAD tags by selecting those with at least eight instances in one population and none in the other. Polymorphism markers can occur through disruption to a cut-site, leading to differential isolation, or through SNPs within the sequence reads of tags that are present in both individuals. We identified 1,136 RS-specific and 1,097 BP-specific RAD markers at 1,890 loci, recapitulating the marker density from our original study using RAD microarrays [11]. SNPs in the sequence read were found in 60.8% of RAD markers and 39.2% had disruptions to the cut site.

DNA from RS-by-BP recombinant *F_2_* individuals was pooled according to lateral plate phenotype. RAD tags were isolated from each pool and sequenced. The 660,000 low-plate pool and 590,000 complete plate pool RAD tags were matched and tabulated at the polymorphic RAD marker sites identified between the RS and BP parents. We previously showed that the lateral plate phenotype inheritance is Mendelian, with the complete-plate phenotype dominant to low-plate [15]. Surprisingly, we also showed that the Mendelian inheritance involves a broad region of LGIV with several distinct areas of linkage. We therefore expected that linked markers in the low-plate *F_2_* pool should be almost exclusively from the BP parent, while the high plate pool, with heterozygous and RS-homozygous individuals, should show a strong bias towards markers from the RS parent. We determined linkage by counting completely associated markers within a bin of contiguous markers. Confirming our previous RAD microarray results, we found three regions of linkage on LGIV, one near the *Eda* locus located at 13 Mb, another at 20 Mb and a third at 25 Mb (Fig. 2B). These data support the utility of RAD tag sequencing for genetic mapping, confirm our previous conclusion that lateral plate variation involves complex genetics, and demonstrate the generality of this finding by showing that the phenomenon exists in multiple Alaskan lake populations.

### Identifying linked markers without a reference genome

We reanalyzed the RAD tag sequence data from above without taking advantage of any available reference genome information. We found eighteen tags completely linked to the lateral plate phenotype. When the genomic locations were examined, all eighteen were spread across the three clusters on LGIV, confirming that Illumina sequencing of RAD tags would provide useful markers for follow-up analyses even in organisms without a reference genome.

### Fine mapping using barcoded adapters

To more closely map the genetic basis of lateral plate loss, we altered two aspects of the RAD tag library preparation. First, *EcoR*I, instead of *Sbf*I, was used to create RAD tags. The more frequent recognition site of *EcoR*I (GAATTC) allowed detection of a greater number of SNPs and thus allowed for higher resolution mapping. Second, we tracked the genotype of each individual in the *F_2_* population by performing separate digestions and ligations to uniquely barcoded P1 adapters for each *F_2_* fish. This enabled all samples to be combined for all subsequent steps of RAD library preparation and sequencing. Pooling and tracking by barcodes eases isolation of RAD tags from many samples and reduces preparation variation between samples.

Sequences from 1.5 million BP and 2.5 million RS parental RAD tags were mapped to 148,390 *EcoR*I cut-site flanking sequences, yielding 2,311 BP-specific and 4,530 RS-specific RAD markers. A total of 3 million and 3.7 million reads were obtained from the low-plate and complete-plate *F_2_* pools, respectively, yielding an average of 16 or 20 RAD tags per polymorphic marker. Thus, each *F_2_* individual had only a subset of RAD markers sequenced. Linked regions were identified by *in silico* pooling and binned frequency counts of linked markers were determined as described above. As with *Sbf*I, *EcoR*I RAD markers identified the same three loci on LGIV, although with a greater number of markers in each region (Fig. 2C). A variable number of sequence reads were obtained from each individual due to varying DNA concentration in the initial samples (Fig. 2D).

Next, the genotypes along LGIV were examined on an individual basis, allowing identification of informative recombinant chromosomes that had breakpoints close to the linked regions (Fig. 2E). To the left of the *Eda* region (12.8 Mb), we found low-plate individuals with RS-specific markers located as close to *Eda* as 12.5 Mb. To the right of *Eda*, we found individuals with RS-specific markers located as close to *Eda* as 14 Mb, defining a region of complete linkage on LGIV in a region less than 1.5 Mb in size surrounding the previously mapped *Eda* locus, corresponding well with the resolution anticipated from an *F_2_* mapping family of this size. Furthermore, by tracking individuals in the low-plate population we determined that the two additional regions had strong, but not complete linkage.

### *In silico* mapping of additional traits segregating within a population

Besides facilitating fine-scale mapping regions identified through bulk segregant analysis, use of individual-specific barcodes also allows mapping of additional phenotypes that are segregating in the cross without additional experimental work. To illustrate this capability, we regrouped the RAD markers used to map the reduction in lateral plate into new phenotypic *F_2_* pools based upon pelvic structure variation (Fig. 2A, arrow). The *in silico* pools of the *F_2_* markers were examined for linkage using binned frequency counts. We were able to map this second trait to the distal tip of linkage group VII (Fig. 2F), confirming previous mapping results [15], [18]. To ask whether this pattern of linkage could occur by chance we used *in silico* re-pooling to determine how often artifactual regions of linkage occur randomly. Monte Carlo simulations were used to populate the pelvic structure pools with random *F_2_* individuals, and the number of markers that passed our scoring thresholds was assayed. Less than 1.3% of simulations had markers scored as linked, and only one simulation (*P* = 0.0007) had three markers map to a single chromosome. None of the simulations had seven contiguous markers as found for the actual pelvic structure mapping. Thus, multiplexed sequencing of a large population allows rapid mapping of multiple traits when genotypes are assayed for each individual.

### Mapping an induced mutation in *N. crassa*

**Figure 3:**
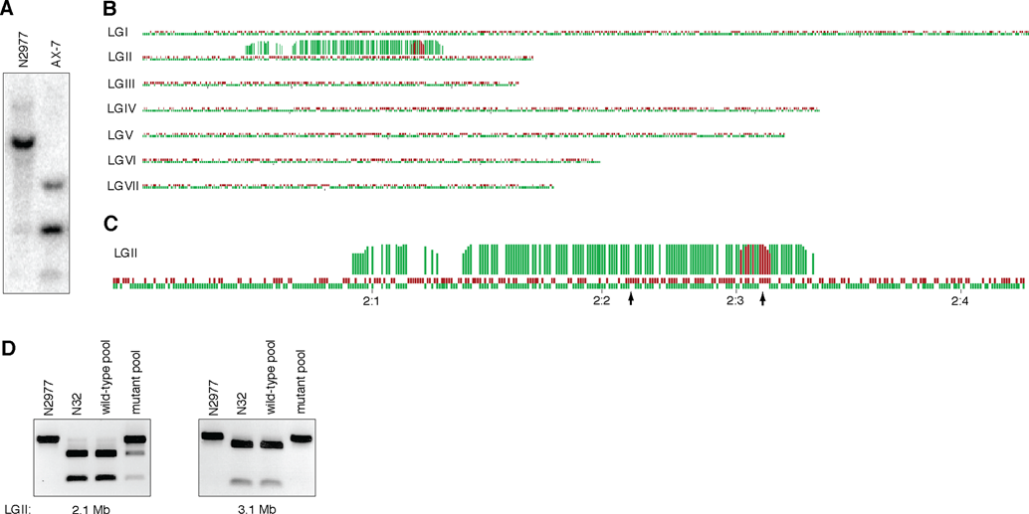
Mapping a novel induced mutation. (A) Southern blot of digested *N. crassa* genomic DNA with methylation-sensitive *Ava*II, showing loss of methylation in the AX7 mutant strain, as compared to the parental mutagenized N2977 strain. (B) Polymorphic RAD markers from N32 (red) and N2977 (green) parental strains were mapped along the seven *N. crassa* linkage groups. Red and green bars above the linkage groups are measures of linkage in the recombinant progeny, indicating the number of tightly linked markers in the local region. (C) A close-up view of linkage group II, showing the locations of confirmative RFLP markers (arrows). (D) RFLP marker confirmation. RFLP markers were designed using polymorphic RAD markers at 2.1 Mb and 3.1 Mb on LGII for *N. crassa*. The marker at 2.1 Mb confirmed the lack of recombinants in the wild-type pool, while a portion of individuals in the mutant pool have undergone recombination at this location. RFLP analysis at 3.1 Mb showed complete linkage in both wild-type and mutant pools to the methylation-deficient phenotype.

Another powerful aspect of the sequenced RAD mapping approach is the ease of SNP discovery, which requires less than two days for library preparation. In parallel with stickleback mapping, we demonstrated the ease of this technique by mapping an induced mutation in a second organism, the fungus *N. crassa*. A methylation-deficient strain, AX7, was derived from the Oak Ridge N2977 background strain (Fig. 3A). We sequenced 1.5 million RAD tags from strain N2977 and the polymorphic Mauriceville strain, N32, which was used in the outcross to create the recombinant mapping population. The sequenced RAD tags mapped to more than 21,000 loci in the reference genome, yielding 2,804 N2977-specific markers and 1,801 N32-specific markers. We pooled and sequenced progeny based on the methylation phenotype and used a binned frequency count analysis that identified significant linkage to ∼122 Kb of contig 5 (located near 3 Mb of LGII as previously assembled [13]) in both pools and a larger region of linkage in the wild-type pool (Fig. 3B,C). We designed restriction fragment length polymorphism (RFLP) markers from RAD markers at 1 Mb of contig 5 (2.1 Mb of LGII) and 0.1 Mb of contig 5 (3.1 Mb of LGII)(Fig. 3C, arrows) and confirmed the linkage pattern in both pools (Fig. 3D). The reason for suppression of recombination in the wild-type pool is unknown.

## Discussion

We have combined RAD marker isolation with massively parallel, high-throughput Illumina sequencing to develop a genotyping platform that can rapidly and cost-effectively discover novel SNP markers and simultaneously genotype many individuals. We demonstrated the feasibility of this platform by discovering more than 13,000 polymorphic markers in two different organisms and by rapidly mapping three traits in two bulk segregant populations and 96 individuals, using less than half the capacity of one run on an Illumina Genome Analyzer. First-generation RAD marker analysis using microarrays has proven to be an effective method for mapping genomic alterations in various species. However, the technique suffers from the need to make species-specific arrays for each organism and perform a new array hybridization for every desired comparison. Our new RAD marker sequencing method adapted the positive aspects of the RAD array approach for high-throughput Illumina sequencing and allows researchers to perform the equivalent of hundreds of RAD array experiments in a single sequencing run. Major benefits of this new approach are a significant increase in the number and type of markers assayed, a decrease in cost and increase in the speed of analyses. In addition, whereas array-based approaches only assay RAD tag presence variation, Illumina sequencing simultaneously provides data on SNPs located outside the recognition site of the restriction enzyme. The ability to sequence heterogeneous DNA samples with the Illumina sequencing platform, along with our sample barcoding, permits multiplexing of experiments such that many individual mapping projects can be performed in parallel, reducing the cost and effort needed to initiate and complete a mapping project.

SNP discovery in a mapping cross requires a high number of sequence reads per marker for each parent. However, *F_2_* individuals in organisms with a reference genome can be lightly sequenced to make genotypic inferences about a genomic region because genetic material is inherited in large blocks defined by recombination breakpoints. We demonstrated this by inferring the genotype of unsequenced markers on LGIV from flanking RAD tag sequences to establish linkage blocks and fine-map the lateral plate breakpoints near *Eda*. A useful two-step approach is to first undersample a high density of markers to identify individuals with informative recombination breakpoints and then acquire additional sequences for these individuals. This ability to infer genotypes in undersequenced large populations allows effective mapping of quantitative trait loci (QTLs) in organisms with sequenced genomes. QTL analyses are most effective when the parental origin of genomic regions in each individual of an *F_2_* mapping family can be determined.

Without a sequenced genome the above strategy of inferring genotypes from neighboring RAD markers will not work. However, we have shown that bulk segregant analysis can successfully identify completely linked markers. Several routes exist for moving from RAD markers to genomic location in organisms without a reference genome. The segregation of RAD markers could be analyzed in a mapping cross to produce a genetic linkage map. In addition, paired-end RAD tag sequences could be used to produce probes or PCR primers to screen fosmid or BAC libraries to identify genomic regions linked to the phenotype.

The RAD marker approach has the flexibility to assay different numbers of markers depending on the choice of restriction enzyme, as we showed by sequencing markers derived from restriction enzymes that have either a 6 or 8 nucleotide recognition sequence. Matching the GC content of a restriction site to a genome can also be used to influence marker number. *EcoR*I (25% GC) had fewer sites in the stickleback genome than expected by chance, while *Sbf*I (75% GC) had greater than the number of sites expected by chance.

With decreasing cost and increasing capacity in sequencing technology, complete sequencing of each individual of interest to determine entire genomic sequences may be a viable option in the future. However, information from contiguous SNPs will often be highly redundant and the additional information gathered at such great density would be wasteful. By focusing sequencing efforts only on those tags flanking a restriction site in multiplexed samples, our novel approach provides significant data complexity reduction and increased throughput. This allows efficient, high-density SNP discovery and genotyping of mapping crosses. Therefore, even as sequencing technology continues to improve, RAD marker sequencing will remain a useful and cost-effective tool for most genetic mapping studies.

## Methods

### Isolation of RAD markers for Illumina sequencing

Genomic DNA (0.1–1 µg; from either individual or pooled samples) was digested for 15 min at 37°C in a 50 µL reaction with 20 units (U) of *EcoR*I or *Sbf*I (New England Biolabs [NEB]). Samples were heat-inactivated for 20 min at 65°C. 2.5 µL of 100 nM P1 Adapter, a modified Solexa© adapter (2006 Illumina, Inc., all rights reserved; for *EcoR*I digestion, top: 5′-AATGATACGGCGACCACCGAGATCTACACTCTTTCCCTACACGACGCTCTTCCGATCTxxxxx-3′ [x = barcode], bottom: 5′-Phos-AATTxxxxxAGATCGGAAGAGCGTCGTGTAGGGAAAGAGTGTAGATCTCGGTGGTCGCCGTATCATT-3′, for *Sbf*I digestion, top: 5′-AATGATACGGCGACCACCGAGATCTACACTCTTTCCCTACACGACGCTCTTCCGATCTxxxxTGCA-3′, bottom: 5′-Phos-xxxxAGATCGGAAGAGCGTCGTGTAGGGAAAGAGTGTAGATCTCGGTGGTCGCCGTATCATT-3′) were added to the sample along with 1 µL of 100 mM rATP (Promega), 1 µL 10× *EcoR*I buffer, 0.5 µL (1000 U) T4 DNA Ligase (high concentration, NEB), 5 µL H_2_O and incubated at room temperature (RT) for 20 min. Samples were again heat-inactivated for 20 min at 65°C, pooled, and randomly sheared (Bioruptor or Branson sonicator 450) to an average size of 500 bp. Samples were then run out on a 1% agarose (Sigma), 0.5× TBE gel and DNA 300 bp to 700 bp was isolated using a MinElute Gel Extraction Kit (Qiagen). The Quick Blunting Kit (NEB) was used to polish the ends of the DNA. Samples were then purified using a Quick Spin column (Qiagen) and 15 U of Klenow exo^−^ (NEB) was used to add adenine (Fermentas) overhangs on the 3′ end of the DNA at 37°C. After another purification, 1 µL of 10 µM P2 Adapter, a divergent modified Solexa© adapter (2006 Illumina, Inc., all rights reserved; top: 5′-Phos-CTCAGGCATCACTCGATTCCTCCGAGAACAA-3′, bottom: 5′-CAAGCAGAAGACGGCATACGACGGAGGAATCGAGTGATGCCTGAGT-3′), was ligated to the DNA fragments at RT. Samples were again purified and eluted in 50 µL. 5 µL of this product was used in a PCR amplification with 50 µL Phusion Master Mix (NEB), 5 µL of 10 µM modified Solexa© Amplification primer mix (2006 Illumina, Inc., all rights reserved; P1-forward primer: 5′-AATGATACGGCGACCACCGA-3′; P2-reverse primer: 5′-CAAGCAGAAGACGGCATACGA-3′), and 40 µL H_2_O. Phusion PCR settings followed product guidelines (NEB) for a total of 18 cycles. Samples were gel purified, excising DNA 300–700 bp, and diluted to 10 nM. Illumina Solexa protocols were followed for sequencing. Sequences are available at the Short Read Archive (http://www.ncbi.nlm.nih.gov/Traces/sra/), at accession SRA001825.1.

### Sequence Analysis

Raw sequence reads were processed by custom Perl scripts (E.A.J.) to optimize read number and reduce artifacts within the data. First, sequences were trimmed to the barcode plus 26 nucleotides to avoid higher error rates at the end of reads while containing enough information to map most reads unambiguously to the reference genome. Sequences with ambiguous ‘N’ nucleotides were eliminated. Reads with identical sequences were grouped by barcode and counted. Polymorphic RAD tags in the parental strains were identified by the presence of at least 8 reads in one strain and none in the other. Polymorphic markers were mapped to the reference genome first identifying perfect matches to potential RAD marker sites in the reference genome and then searching for single mismatches. Polymorphic markers were assessed for inheritance in individual *F_2_* progeny and classified as maternal or paternal. Regions of linkage were identified by binning neighboring markers and counting linked markers within the bins. For studies involving the stickleback *EcoR*I RAD tags, 10 contiguous markers were binned and the numbers of linked markers were counted (i.e. number of markers lacking RS-specific RAD tags in the low-plate population, or having fewer than three BP-specific RAD tags in the high-plate population). For individual tracking, the presence of a RS-specific RAD tag in a low-plate individual was considered sufficient to infer that the region was inherited from the RS parent. Because individual chromosomes generally had single crossover events, the breakpoint between the block of RS-parent chromosome and BP-parent chromosome could be defined by the end of a region containing multiple RS-specific tags. For the bulk-segregant stickleback study, linked markers with fewer than three tags from the RS parent were counted in each bin of 10 markers in the low-plate pool and fewer than three markers from the BP parent were counted in the complete-plate pool. The same binning thresholds were used in the *N. crassa* pools, counting wild-type-specific markers in the mutant pool and mutant-specific markers in the wild-type pool.

### Crosses and DNA isolation

The stickleback fish used were from lines originally derived from wild samples collected in the Matanuska-Susitna Borough of Alaska. A freshwater-derived individual lacking lateral plates and pelvic structure (Bear Paw Lake) was crossed to an oceanic individual possessing the complete form of each phenotype (Rabbit Slough). A full-sib cross between two *F_1_* individuals produced the *F_2_* individuals that were used for the mapping analysis. Crosses, rearing and phenotypic analyses were performed as previously described [15]. Genomic DNA was isolated from pectoral fin clips using a DNeasy Tissue Kit (Qiagen). DNAs from 96 *F_2_* individuals were uniquely barcoded, which allowed us to track RAD markers and associate them with differing lateral plate or pelvic structure phenotypes. *F_2_* individuals used in the mapping analysis included 60 fish possessing the complete lateral plate phenotype, 31 low lateral plate individuals, 67 individuals demonstrating the high pelvic structure phenotype and 29 with a low pelvic score.

To isolate *N. crassa* mutants that are defective in DNA methylation, we mutagenized strain N2977 (*a bar^m^*; *hph^m^*; *Δinl am^132^*), which contains methylated, and therefore, silent copies of basta and hygromycin resistance genes (*bar^m^* and *hyg^m^*, respectively). Following mutagenesis, we selected mutant strains that grew in the presence of both drugs. Subsequent Southern analysis revealed that the selected strains displayed global defects in DNA methylation patterns. The original mutant isolates were backcrossed to strain N3311 (*A sad-1*; *bar^m^*; *hph^m^*; *Δinl am^132^*) to isolate homokaryons and to confirm that the mutant phenotype was caused by a mutation in a single gene. To generate recombinant progeny, the AX7 mutant was crossed to the polymorphic Mauriceville wild-type strain (N32) and 28 mutant progeny and 24 wild-type progeny were isolated. For genotyping, RAD tags were prepared from genomic DNA from background parental strains N32 and N2977, as well as mutant and wild-type progeny pools from the N32 and AX7 cross. The RFLP primers used were as follows: RFLP2.1, forward: 5′-GCCTTTTGCTCTACTCGCATCG-3′, reverse: 5′-TCCGCAGTCTTTGGGTTACGAC-3′; RFLP3.1, forward: 5′-TTGATGTGGAGTCTTCGCCG-3′, reverse: 5′-GCTTTGCTCGTCATTTGGGTC-3′. *EcoR*I was used to digest the RFLP markers.
